# Enterohemorrhagic *Escherichia coli* promotes the invasion and tissue damage of enterocytes infected with *Candida albicans* in vitro

**DOI:** 10.1038/srep37485

**Published:** 2016-11-22

**Authors:** Weiming Yang, Yanjun Zhou, Chunrong Wu, Jianguo Tang

**Affiliations:** 1 Department of Trauma-Emergency & Critical Care Medicine, Shanghai Fifth People’s Hospital, Fudan University, Shanghai 200240, P.R. China; 2 Division of Swine Infectious Diseases, Shanghai Veterinary Research Institute, Chinese Academy of Agricultural Sciences, Shanghai, 200241, P.R. China

## Abstract

The principal aim of this study was to investigate the *in vitro* co-infection of Caco-2 cells with *Candida albicans* and enterohemorrhage *Escherichia coli* (EHEC). The ability of both species to colonize or invade the Caco-2 cells was evaluated by indirect immunofluorescence and inverted microscopy. The damage to Caco-2 cells was evaluated by measuring lactate dehydrogenase (LDH) activity. *C. albicans* virulence gene expression (*HWP1*, *ALS3*, *PLB1*, *SAP4*, and *EFG1*) was evaluated by quantitative real-time polymerase chain reaction (qRT-PCR). Compared to single infections with enterohemorrhage *Escherichia coli* or *C. albicans*, a co-infection colonized or invaded Caco-2 cells more quickly, and *C. albicans* tended to accumulate more easily, accompanied by the upregulation of related genes. In addition, the LDH activity in the co-infected group was higher than in cells infected with *C. albicans* or with enterohemorrhage *Escherichia coli*, accompanied by the upregulation of toxicity-related genes. Using Caco-2 cells as an infection model, this study demonstrated that co-infecting *in vitro* enterocytes with *C. albicans* and enterohemorrhage *Escherichia coli* enhanced the invasiveness and tissue damaging effects of *C. albicans*.

As opportunistic fungal pathogens, *Candida* species are normally harmless commensals in the gastrointestinal tract, genitourinary tract or oropharyngeal tract of most healthy individuals^1^. However, in specific conditions, such as long-term antibiotic treatment, immunocompromisation or critical illness, *Candida* species can also turn into pathogens and cause superficial infections, such as oral or vaginal candidiasis, deep-seated infections, and systemic infections^2^,^3^.

The main pathogenic *Candida* species in the human body is *C. albicans*^4^,^5^, notably the *C. albicans* in gastrointestinal tract. *C. albicans* can not only cause intestinal candidosis, but can also destroy the intestinal epithelial barrier and result in the occurrence of candidemia^6^,^7^. However, most common clinical situations involve a co-infection by various microorganisms, rather than a single infection by one microorganism. In recent years, there have been reports on the effects of co-infection leading to invasive candidosis^8^,^9^,^10^,^11^,^12^. Several studies have reported the enhanced adherence and invasiveness of *C. albicans* on epithelium cells, as well as the tissue damage caused by *C. albicans* during co-infection^8^,^11^,^12^,^13^,^14^,^15^. Most of these studies focused on co-infection by various *Candida* species; there are few studies focused on co-infection by *Candida* species and a non-*Candida* species^13^,^16^,^17^.

The aim of this study was to characterize a *C. albicans* and enterohemorrhage *Escherichia coli* co-infection on enterocytes *in vitro* using Caco-2 cells. Indirect immunofluorescence and quantitative real-time polymerase chain reaction (qRT-PCR) was used to evaluate results. The expression of virulence genes (*HWP1*, *ALS3*, *PB1*, *SAP4* and *EFG1*) was also analyzed.

## Results

### Colonization or Invasion of Caco-2 cells by *C. albicans*

The positive rates of detection in the single infection group and the co-infection group across all time points are summarized in Table 1. Results indicated that the positive rates in the co-infection group were significantly higher than those in the single infection group within the first hour after infection. At later time points, no statistically significant differences were detected between the groups.

**Figure 1:**
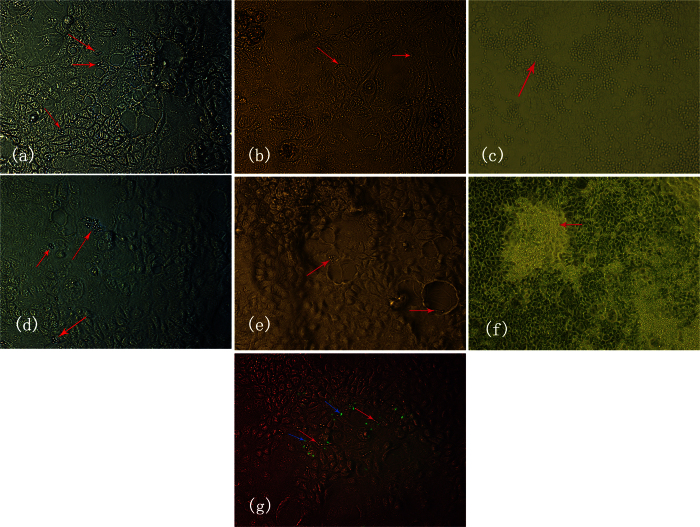
The morphogenic states of C. albicans during infection in the single infection group (**a**–**c**) and the co-infection group (**e**–**f**) at different observation time points (A 1 hour, B 2 hours, C 3 hours, D 0.5 hours, E 2 hours, F 3 hours). Green fluorescent dots (blue arrows) indicate enterohemorrhage *Escherichia coli* and red arrows indicate C. albicans in the co-infection group (**g**). Magnification: 10 × 20.

Under inverted microscopy, in the single infection group (group 1), *C. albicans* were detected at the yeast phase, pseudohyphal phase and hyphal phase from the time point of 1 hour onward (Fig. 1a), 2 hours onward (Fig. 1b) and 3 hours onward (Fig. 1c) after infection, respectively. In the co-infection group (group 3), *C. albicans* were detected at the yeast phase, pseudohyphal phase and hyphal phase from the time point of 30 minutes onward (Fig. 1d), 2 hours onward (Fig. 1e) and 3 hours onward (Fig. 1f) after infection, respectively. *C. albicans* tended to cluster more often in the co-infection group than in the single infection group. In addition, enterohemorrhage *Escherichia coli* and *C. albicans* were present in the same location in the co-infection group (Fig. 1g).

### LDH activity as an indicator of tissue damage

**Figure 2:**
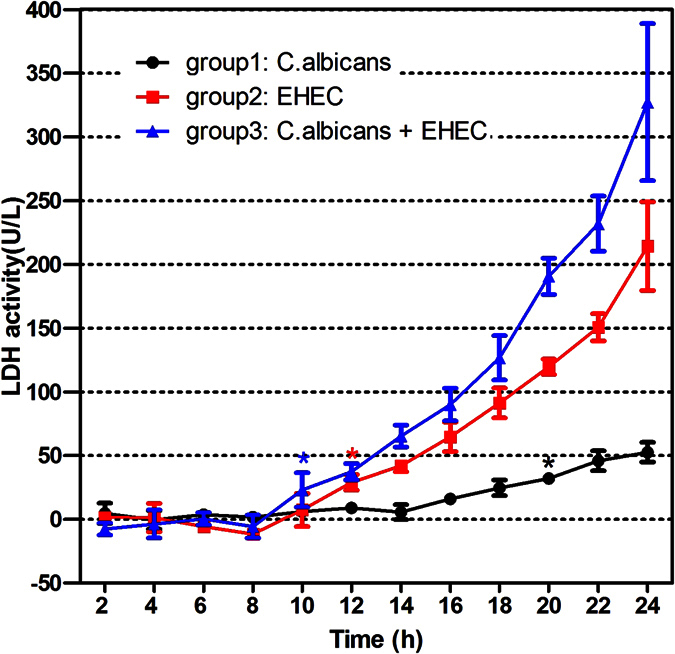
LDH activity at different time points (black asterisk: 20 hours; red asterisk: 12 hours; blue asterisk: 10 hours). *Significantly higher than the corresponding preoperative level (*P* < 0.05).

In order to determine the extent of Caco-2 cell damage induced by *Candida*, the levels of LDH activity were measured every 2 hours for 24 hours after infection. It is important to highlight that half of the number of *Candida* cells and enterohemorrhage *Escherichia coli* cells were present in the starting inoculum of the co-infection group. Repeated measures analysis of variance was used to test if the measurements were equal between each time point (the measurement at the first time point was taken as the control). The results suggested that LDH values in group 1, 2, and 3 increased with time. LDH values in group 1, 2 and 3 significantly increased at 20 hours, 12 hours and 10 hours, respectively (Fig. 2). When comparing the total trends among the three groups, it was found that LDH values in group 2 were greater than those in group 1, taking all measurements as a whole (*P* < 0.001), group 3 greater than in group 1 (*P* = 0.0004), and group 3 greater than in group 2 (*P* = 0.0028). These results indicate that the tissue damage in the co-infection group was greater than the damage of a single infection.

### Gene expression

qRT-PCR revealed a wide range of expression of toxicity-related genes. The results were expressed as the mean percentage expression, relative to the expression of *ACT1* during Caco-2 cell infection. *ACT1* gene expression levels were constant in all assays.

**Figure 3:**
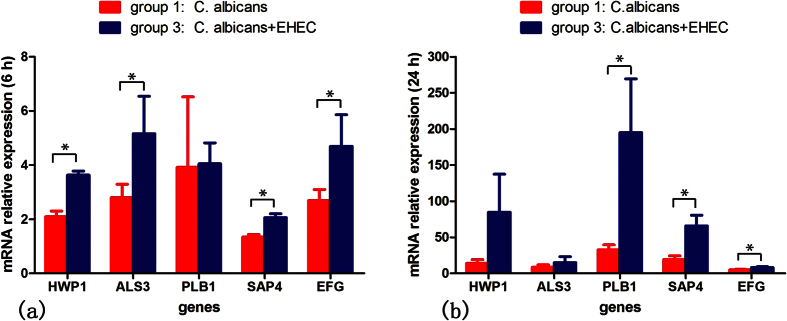
mRNA relative expression at 6 hours (**a**) and 24 hours (**b**) after infection. *Significantly different between groups (*P* < 0.05).

*HWP1* and *ALS3* expression by *C. albicans* was upregulated during co-infection with enterohemorrhage *Escherichia coli* 6 hours post-infection (*P* = 0.0005, *P* = 0.0491) (Fig. 3). Expression of both *PLB1* and *SAP4* genes was detected 6 and 24 hours after infection in both groups. *PLB1* gene expression was upregulated in the co-infection group at 24 hours after infection (*P* = 0.0193) (Fig. 3), but *SAP4* gene expression was upregulated in the co-infection group at both 6 and 24 hours after infection (*P* = 0.0019, *P* = 0.0062) (Fig. 3). *EFG1* gene expression demonstrated similar results to *SAP4* expression: an upregulation in the co-infection group at both 6 and 24 hours post-infection (*P* = 0.0473, *P* = 0.0227) (Fig. 3).

## Discussion

The intestinal mucosal barrier is highly important in host defense, as it is the first cell layer that prevents an invasion of pathogens, such as *Candida* species and enterohemorrhage *Escherichia coli*^18^. In recent years, a number of studies have demonstrated that the gastrointestinal tract is the main reservoir for *C. albicans* and a source of gastrointestinal candidiasis and candidemia^6^,^7^. It was found in our study that enterohemorrhage *Escherichia coli* promotes the colonization and invasiveness and tissue damaging effects of *C. albicans* to enterocytes *in vitro*.

The invasion of epithelial cells by *C. albicans* is a key element in the physiopathology of candidiasis. It is generally accepted that yeast are the noninvasive, commensal morphology^19^,^20^. However, in our study, we discovered that yeast-phase *C. albicans* could colonize or invade Caco-2 cells in the early stages of both single and co-infections. However, the colonization or invasion of Caco-2 cells by *C. albicans* occurred earlier when combined with enterohemorrhage *Escherichia coli* than it did during a single infection. Two other observations were that enterohemorrhage *Escherichia coli* and *C. albicans* were present in the same location in the co-infection group and that the co-infection group tended to cluster more often than the single infection group. This suggests that *C. albicans* colonized or invaded enterocytes more efficiently in the co-infection group. It is interesting to note that there were no differences between the single infection and co-infection groups when it came to the presence of the pseudohyphal and hyphal phases. Although the underling mechanism remains unclear, one possible explanation is that enterohemorrhage *Escherichia coli* promotes the superficial infection of Caco-2 cells with *C. albicans* by inducing damage to the epithelium. This would facilitate the access of *C. albicans* to the epithelial layers.

The invasion of host cells by *C. albicans* is associated with expression of the hyphal-associated genes. *ALS3* is a hyphal-specific gene expressed by *C. albicans* hyphae and pseudohyphae, but not yeast^21^,^22^. Both *ALS3*^21^,^22^,^23^ and *HWP1*^23^,^24^ were directly associated with hyphae formation and invasiveness in *C. albicans*. In this experiment, qRT-PCR showed that during co-infection, *C. albicans* strains exhibited an upregulation of *HWP1* and *ALS3* compared to a single infection at 6 hours post-infection. Based on the qRT-PCR results and the functions of *ALS3* and *HWP1*, it is tempting to speculate that invasiveness of *C. albicans* in the co-infection group is enhanced by the presence of *C. albicans* hyphae following *HWP1* and *ALS3* upregulation.

LDH activity was used to evaluate the degree of tissue damage in the single infection and co-infection groups. Of the single species infection groups, enterohemorrhage *Escherichia coli* caused more damage than *C. albicans*. Compared to group 1, in group 2, tissue damage was more severe and occurred earlier. This result was expected, given the fact that enterohemorrhage *Escherichia coli* is deemed more pathogenic. In the co-infection group, the degree of damage was more severe than both groups 1 and 2. These results suggest that tissue damage in the co-infection group was greater than the damage of a single infection. According to these findings, it is obvious that potential synergism may exist between microorganisms in a co-infection that enhances damage caused to epithelial cells.

In order to better elucidate pathogenic differences, *PLB1* and *SAP4* genes expressions were measured. *PLB1* is considered an important virulent factor in intestinal *C. albicans* bacteremia^25^. Expression of this enzyme can lead to the disruption of membrane structure and function^26^. The SAP gene family was also confirmed to play a crucial rule in the virulence of *C. albicans*^27^. The current study showed that the expression of *PLB1* and *SAP4* genes was always detected at 24 hours after infection, confirming a potential role of these genes in enterocyte cell damage. In addition, *PLB1* and *SAP4* were expressed at a higher level in the co-infection group than in the single infection groups, indicating that enterohemorrhage *Escherichia coli* promotes the tissue damaging effects of *C. albicans*, with the presence of extracellular hydrolytic enzymes following *PLB1* and *SAP4* upregulation.

It is well established that *EFG1* and components of its upstream regulatory pathway are essential for all stages of *C. albicans*-epithelial interaction^23^. In the present study, the EFG expression of *C. albicans* by qRT-PCR was measured both in the single infection and co-infection groups. The results showed that EFG expressions at both 6 and 24 hours after infection were greater in the co-infection group than in the single infection group, indicating that enterohemorrhage *Escherichia coli* promoted the ability of *C. albicans* to invade and cause tissue damage to Caco-2 cells.

It should be noted that the inverted microscope and the immunofluorescence methodology used in the present study cannot discriminate between *C. albicans* that are adherent to Caco-2 cells versus ones that have invaded Caco-2 cells.

In summary, the present study confirms the effectiveness of Caco-2 cells as an *in vitro* model to study the attributes of *Candida* virulence. Usage of indirect immunofluorescence, LDH activity evaluation, and the developed methodology of RT-PCR for *Candida* cell quantification in tissue, has conclusively shown that co-infecting *in vitro* enterocytes with *C. albicans* and enterohemorrhage *Escherichia coli* enhanced the invasiveness and tissue damaging effects of *C. albicans*.

## Material and Methods

### Microorganisms

Enterohemorrhage *Escherichia coli* O157:H7 and *C. albicans* ATCC 10231 (obtained from the American Type Culture Collection) were used for all assays in this study. The identity of the *C. albicans* strains were confirmed by PCR-based sequencing using specific primers ITS1 (5′-TCCGTAGGTGAACCTGCGG-3′) and ITS4 (5′-TCCTCCGCTTATTGATATGC-3′)^28^.

*C. albicans* strains were routinely cultured in liquid YPD (1% yeast extract, 2% bacto-peptone, 2% D-glucose) medium in a shaking incubator at 120 rev/min for 48 hours at 37 °C, and maintained in solid YPD (2% agar was added to liquid YPD). Enterohemorrhage *Escherichia coli* strains were cultured in LB (0.5% yeast extract, 1% tryptone, 1% NaCL) broth at 37 °C for 10 hours.

Both microorganisms were measured with the viable plate count method. After this, the cellular densities of *C. albicans* and *E. coli* were adjusted to 2 × 10^6^ cells/ml before the experiment. *C. albicans* cells were grown to log phase for another 2 hours at 37 °C prior to usage.

### Culture of intestinal epithelial cells

The colon adenocarcinoma-derived cell line Caco-2 (obtained from the Insitute of Biochemistry and Cell Biology, Shanghai, China) was used as an *in vitro* model for intestinal cells in this study. Caco-2 cells are capable of forming monolayer cells that are similar, in many ways, to normal human enterocytes. These cells were routinely cultured in Dulbecco’s modified eagle medium (DMEM), supplemented with 10% fetal calf serum (FCS), without antibiotics or antifungal agents. The cells were maintained in a humidified incubator at 37 °C in 5% CO_2_ environment in saturated humidity. For every experiment, 1 × 10^5^ Caco-2 cells were seeded onto each well of a 12-well plate and cultured up to a high cell density before the experiment.

### Groups and infection

Four groups were assigned to study *in vitro* single infection and co-infection of Caco-2 cells by *C. albicans* and enterohemorrhage *Escherichia coli*. In group 1, Caco-2 cells were infected with 1 ml 2 × 10^6^ *C. albicans* cells. In group 2, Caco-2 cells were infected with 1 ml 2 × 10^6^ enterohemorrhage *Escherichia coli* cells. These two groups, 1 and 2, were single species infections. In group 3, Caco-2 cells were infected with both 0.5 ml 2 × 10^6^ *C. albicans* cells and 0.5 ml 2 × 10^6^ enterohemorrhage *Escherichia coli* cells. Group 4 was set as a negative control and neither infected with *C. albicans* nor enterohemorrhage *Escherichia coli*. All groups were incubated at 37 °C in a 5% CO_2_ environment in saturated humidity.

### The surface colonization or invasion of Caco-2 cells by *C. albicans*

The *in vitro* adherence assay was performed according to a previously described protocol^29^. The surface colonization or invasion of Caco-2 cells by *C. albicans* in single and co-infections was determined using inverted microscopy and indirect immunofluorescence. Briefly, the Caco-2 cells were infected with *C. albicans* and enterohemorrhage *Escherichia coli*. Every 30 minutes after infection, the medium above the cells was aspirated and the monolayers were rinsed three times with phosphate buffer solution (PBS) to remove fungal cells not associated with epithelial cells, followed by fixation in 80% alcohol for 40 minutes at 4 °C. All fungal cells remaining that had colonized or invaded the surface of the Caco-2 cells were determined directly by inverted microscopy. Once the presence of C. albicans was observed, the colonization or invasion of Caco-2 cells by *C. albicans* was considered positive. The experiments were repeated twelve times at each time point. The positive rate at each time point was calculated as the number of positive detections divided by 12 experiments. Fisher’s exact test was used to examine the differences in the positive rate between the single infection and co-infection groups. Enterohemorrhage *Escherichia coli* was stained by Anti-*E. coli* O157 antibody (FITC) (abcam). After rinsing with PBS three times, each well was fully observed under indirect immunofluorescence in sequences to ensure all fields were observed.

### Damage assay: Lactase dehydrogenase (LDH) activity

The extracellular activity of LDH released from infected enterocytes into the medium was monitored as a measure of tissue damage. A Cytotoxicity Detection Kit (LDH) (Roche Diagnostics, Indianapolis, IN) and Modular P800 (Roche) were used for the damage assay. Arguments of Modular P800 were set as follows: Assay-Rate A, Wavelength-492/690 nm, time-10 mins, point- 22~32.

The extracellular LDH activity of all groups was continuously measured at 2, 4, 6, 8, 10, 12, 14, 16, 18, 20, 22 and 24 hours after infection. The degree of tissue damage was defined as the LDH activity of experimental groups (groups 1, 2, and 3) subtracting the LDH activity of the control group, which was incubated with growth medium alone (group 4). The experiments were performed three times.

### Analysis of fungal virulence gene expression

#### RNA extraction

For RNA extraction, samples were collected in a 1.5 ml microcentrifuge tube and frozen by dipping in liquid nitrogen, followed by adding 500 μl Buffer RB/ ß-mercaptoethanol. Water-saturated phenol, 2 M NaAc (pH 4.0), and chloroform were added to the mixture in order to lyse cells. Fungal RNA extraction kit (E.Z.N.A. ^®^ Fungal RNA Kit, Omega) was then used to complete total RNA extraction from the tissue, according to the manufacturer’s recommendations. The procedure was performed twice.

### Synthesis of complementary DNA (cDNA)

For synthesis of cDNA, PrimeScript^®^RT Master Mix (Takara) was used to synthesize cDNA, following the manufacturer’s recommendations with a modification. Briefly, 8 μl RNA was incubated with 2 μl 5X PrimeScript RT Master Mix at 37 °C for 15 minutes. Then, the mixture was placed at 80 °C for 10 seconds to stop the reaction of synthetase.

### Primer design

The following pairs of primers were used in this assay: *ACT1*^30^, *EFG1*^30^, *HWP1*^31^, *ALS3*^32^, *SAP4*^33^, and *PLB1*^13^ (Table 2). The *ACT1* had been previously used as a housekeeping gene and was also used in this study^30^. To verify the specificity of each primer pair for its corresponding target gene, PCR using the various primer pairs was applied to genomic DNA extracted from each of the *Candida* strains. The mRNA expression levels of these selected genes were measured to evaluate the impacts of co-infection.

### Quantitative real-time PCR (qRT-PCR) assay

qRT-PCR was performed using SYBR Premix Ex Taq (Takara). A 20 μl mixture solution was composed of 10 μl SYBR Premix Ex Taq, 2 μl primers, 1 μl cDNA and 7 μl deionized water. qRT-PCR was performed with an initial denaturation step at 94 °C for 30 seconds, followed by 40 cycles of denaturation at 95 °C for 5 seconds, and primer annealing at 60 °C for 34 seconds. A melting curve was generated at 95 °C for 15 seconds, 60 °C for 1 minute and 95 °C for 15 seconds at the end of each PCR cycle to verify that a specific product was amplified. Control samples were included on each plate to ensure that multiple plates could be compared. All samples were run in triplicate. The Ct value of each sample was determined, and the relative gene expression levels were calculated using the ΔΔCt method, which was normalized to the housekeeping genes described above, as a control.

### Statistical analysis

Data were analyzed using the SAS 9.4 statistical software (SAS Institure, Inc., Cary, NC). Continuous variables were expressed as the mean ± standard deviation while categorical variables were expressed as frequency and percentage. The independent Student’s t test was used to compare mRNA relative expression. Fisher’s exact test was performed to compare the positive detection rate of *C. albicans* between group 1 and group 3. Repeated measures analysis of variance was used to compare the LDH values within and between groups by taking different times for measurement as the repeated factor. Statistical significance level was set at 0.05.

## Additional Information

**How to cite this article**: Yang, W. *et al*. Enterohemorrhagic *Escherichia coli* promotes the invasion and tissue damage of enterocytes infected with *Candida albicans* in vitro. *Sci. Rep*. **6**, 37485; doi: 10.1038/srep37485 (2016).

**Publisher's note:** Springer Nature remains neutral with regard to jurisdictional claims in published maps and institutional affiliations.

## Figures and Tables

**Table 1:** Positive detection rates between single infection and co-infection groups across time points.

Time points	positive rates		P value
	Group 1	Group 3	
0.5 h	0%	42%	0.0186
1 h	33%	83%	0.0167
1.5 h	75%	92%	0.2484
2 h	100%	100%	
2.5 h	100%	100%	
3 h	100%	100%	

Group 1: Caco-2 cells infected with 1 ml 2 × 10^6^ *C. albicans* cells. Group 3: Caco-2 cells infected with both 0.5 ml 2 × 10^6^ *C. albicans* cells and 0.5 ml 2 × 10^6^ enterohemorrhage *Escherichia coli* cells. h: hours.

**Table 2:** Forward (FW) and reverse (RV) primers used for real-time PCR.

Sequence (5′-3′)	Orientation	Target
TTT CAT CTT CTG TAT CAG AGG AAC TTA TTT	Forward	*ACT1*^30^
ATG GGA TGA ATC ATC AAA CAA GAG	Reverse	
ACG TGG TAG AAG AGA TGG GA	Forward	*EFG1*^30^
TGC ATT AGG AGT TAC TCC GG	Reverse	
CAG AAG CTT CCA TTC CAC CT	Forward	*HWP1*^31^
TTT GGA ACA GCT GGA GAG GT	Reverse	
CAA CTT GGG TTA TTG AAA CAA AAA CA	Forward	*ALS3*^32^
AGA AAC AGA AAC CCA AGA ACA ACC T	Reverse	
CAA TTT AAC TGC AAC AGG TCC TCT T	Forward	*SAP4*^33^
AGA TAT TGA GCC CAC AGA AAT TCC	Reverse	
GCT CTT TTC AAC GAA GCG GTG T	Forward	*PLB1*^13^
GCC ATC TTC TCC ACC GTC AAC T	Reverse	
